# Internet-based cognitive-behavioral therapy for premenstrual syndrome: a randomized controlled trial

**DOI:** 10.1186/s12905-021-01589-7

**Published:** 2022-01-08

**Authors:** Sanam Borji-Navan, Sakineh Mohammad-Alizadeh-Charandabi, Khalil Esmaeilpour, Mojgan Mirghafourvand, Ali Ahmadian-Khooinarood

**Affiliations:** 1 Student Research Committee, Tabriz University of Medical Sciences, Tabriz, Iran; 2 Social Determinants of Health Research Centre, Department of Midwifery, Faculty of Nursing and Midwifery, Tabriz University of Medical Sciences, South Shariati Street, Tabriz, 5137975846 Iran; 3 Department of Psychology, Faculty of Education and Psychology, University of Tabriz, Tabriz, Iran; 4 Clinical Research Development Unit, Imam Reza General Hospital, Tabriz University of Medical Sciences, Tabriz, Iran; 5 Education Development Center, Tabriz University of Medical Sciences, Tabriz, Iran

**Keywords:** Cognitive behavior therapy, ICBT, Internet-based treatment, Premenstrual syndrome, Quality of life

## Abstract

**Background:**

Premenstrual syndrome (PMS) is a common problem of women of reproductive age, affecting various aspects of their lives. However, limited studies have investigated the effect of internet-based cognitive-behavioral therapy (ICBT) on PMS. Therefore, we aimed to assess whether ICBT can reduce symptom severity of women with PMS and improve their quality of life during the perimenstrual and late follicular phases of menstrual cycle.

**Methods:**

The study included 92 university students aged 18–35 years who had moderate to severe PMS. The participants were allocated into two groups of 46 using block randomization. The intervention group underwent ICBT for two menstrual cycles, while the control group received no intervention. Before and after the intervention, all participants filled the Daily Record of Severity of Problems (DRSP) for two menstrual cycles and the Quality of Life Enjoyment and Satisfaction Questionnaire—Short Form (Q-LES-Q-SF) on days 1–2 and 11–13 of the menstrual cycle. Data were analyzed using univariate general linear models.

**Results:**

Four students in the intervention group were lost to follow-up. Following the intervention, the mean score of total PMS symptoms was significantly lower in the intervention group than in the control group (10.4 vs. 20.2, adjusted difference: − 9.9 [95% CI − 13.3 to − 6.6]), and the score of perimenstrual quality of life was significantly higher (64.2 vs. 50.3, 14.1 [8.5 to 19.8]). However, there was no significant intergroup difference in the late follicular quality of life (68.3 vs. 67.3, 1.9 [− 4.4 to 8.1]).

**Conclusions:**

The ICBT could reduce the symptom severity of women suffering from PMS while improving their perimenstrual quality of life. However, it had no significant effect on the late follicular quality of life. Therefore, this intervention can be used for women with PMS.

*Trial registration* The Iranian Registry of Clinical Trials, Identifier: IRCT20100414003706N34, Registered prospectively on 19 June 2019, https://www.irct.ir/trial/38394.

**Supplementary Information:**

The online version contains supplementary material available at 10.1186/s12905-021-01589-7.

## Background

Some women experience several troublesome symptoms during their premenstrual period, which is a condition called the premenstrual syndrome (PMS) [1]. According to the Diagnostic and Statistical Manual of Mental Disorders, 5th edition (DSM-5), any woman experiencing at least five symptoms, including at least one affective symptom, of the PMS criteria within one week before menstruation for two consecutive cycles is suffering from PMS [2]. The symptoms should be severe enough to interfere with the patient's daily life [3].

In general, PMS affects about 20% of women of childbearing age [4, 5]. The condition leads to various consequences, including physical (cardio-pulmonary, genitourinary, neurological, gastrointestinal, and musculoskeletal disorders), psychological (mood, cognitive, and emotional disorders), behavioral (changes in sleep and eating patterns), and social (defective interpersonal relationships, sexual dysfunction, and social isolation) complications [6]. The related symptoms can significantly impact the quality of life of the affected women [7], especially during the premenstrual period [8]. Given the high importance of the quality of life assessment in patients with chronic problems [9], it is necessary to investigate the potential effect of various interventions on the quality of life of women suffering from PMS.

Our understanding of the cause and pathophysiology of PMS is not complete. A great deal of PMS is still unknown to us [10]. This condition is one of the most troublesome and common complaints of women at their visits to healthcare facilities for which there is no definitive cure [11]. Therefore, there is a need for effective and acceptable palliative methods with no or low side effects for women with this problem.

Some studies have reported the effectiveness of cognitive-behavioral therapy on PMS symptoms [12–14]. However, this method is not feasible for some women due to limitations, such as distance and time constraints and its cost-ineffectiveness [15]. We only found one study investigating the effect of internet-based cognitive-behavioral therapy (ICBT) on PMS. The study was conducted in Germany on women aged 18–45 years, reporting promising results [16]. Given the effect of social, cultural, and psychological factors on PMS [17], the results of ICBT interventions in one region cannot be generalized to other areas with different social and cultural backgrounds. Therefore, the present study aimed to investigate the effect of ICBT on symptom severity and quality of life of Iranian female university students suffering from PMS as the primary outcomes. Moreover, the PMS-related disability and attitude toward menstruation were investigated as the secondary outcomes.

## Methods

### Study design and participants

The present randomized controlled trial was approved by the Ethics Committee of the Tabriz University of Medical Sciences with the approval code of IR.TBZMED.REC.1398.014 and was registered prospectively at the Iranian Registry of Clinical Trials with the registration code of IRCT20100414003706N34. Participant recruitment was then conducted. We followed the CONSORT guidelines.

The inclusion criteria were healthy university students aged 18–35 years who lived in the residence halls of the Tabriz University of Medical Sciences (4 residence halls with approximately 1200 students) who had regular menstruation, a menstrual cycle duration of 25–35 days in the past 6 months, had moderate to severe PMS that the diagnosis was confirmed using the Daily Record of Severity of Problem (DRSP) for two menstrual cycles before inclusion into the study, and did not use any treatment for PMS or premenstrual dysphoric disorder (PMDD) currently.

Exclusion criteria were as follows: being a professional athlete, pregnancy, breastfeeding, a history of childbirth within the last year, major depression at the time of the study, a history of a major psychiatric disorder (psychosis, bipolar disorder, eating disorder, and severe depression), taking especial medications (antidepressants, benzodiazepines, anticancer drugs, oral contraceptives, or hormones) within the past three months, being affected with especial diseases (epilepsy or gastrointestinal, cardiovascular, renal, and endocrine disorders) at the time of the study, having experienced a highly stressful event (parental divorce or death of a first-degree family member) during the past 6 months, current or previous gynecological problems (previous hysterectomy, oophorectomy, gynecological cancer, polycystic ovary syndrome, endometriosis, and infertility), no access to the internet and mobile phones, and a history of drug, alcohol, or hookah use within the past two years.

The patients were assessed for major depression using the Beck questionnaire and the DRSP, while other exclusion criteria were applied based on the participants' self-reporting. Moreover, we tried to include some students from two private residence halls (with a total population of 225 students). However, those in charge did not cooperate with us. Therefore, we could not enroll any participants from the private resident halls.

### Sample size

Considering the PMS scores from a previous study conducted in a similar study setting (M1 = 163.6, SD1 = 89.5) [18], an estimated reduction of 35% in the mean PMS score due to the intervention (M2 = 106.3, SD2 = SD1), two-sided α = 0.05, power = 80%, and 15% probability of loss to follow-up, the sample size was set at 92 (n = 46 for each group). This sample size was sufficient to meet other study objectives, with a power greater than 80%.

### Recruitment and randomization

At first, the potential participants, which were selected using a checklist of initial eligibility criteria, completed the socio-demographic and reproductive questionnaire and a validated Persian version of the 19-item Premenstrual Symptoms Screening Tool (PSST) [19]. According to the PSST developers [20], the screening is positive when the woman reports at least five of 14 symptoms related to PMS. The reported symptoms must be moderate or severe, and at least one of them must be included in one of the four main symptoms of PMS. In addition, the symptoms must moderately to severely interfere with at least one of the five domains of life.

All participants with positive screening provided written informed consent. Then, they were asked to complete the DRSP during the following menstrual cycle. Moreover, they were asked to fill the validated Persian version of the 21-item Beck Depression Inventory [21] on one of the days of their mid-follicular phase (days + 7 to + 10 of the menstrual cycle). The participants with a score of 29 or higher on the Beck inventory [22] were diagnosed with major depression and were subsequently excluded from the study. The remaining participants were asked to complete the DRSP during a second menstrual cycle. Moreover, they filled the Sheehan Disability Scale (SDS) and the Menstrual Attitude Questionnaire (MAQ) on day 1 or 2 of the menstruation. Also, they filled the Quality of Life Enjoyment and Satisfaction Questionnaire—Short Form (Q-LES-Q-SF) twice, including one on days 1–2 and once on days 11–13 of the menstrual cycle. Participants with symptoms of major depression in the follicular phase based on the 2-cycle DRSP were also excluded.

The female students with moderate to severe PMS that was diagnosed based on the DRSP were finally included in the study. These participants were randomly allocated into the intervention and control groups using the block randomization method. The allocation sequence was generated with randomly varied block sizes of four and six and an allocation ratio of 1:1 using an online program (www.random.org). The sequence generation was performed by a person not involved in the participant recruitment and data collection. The central telephone method was used for allocation concealment, while participant recruitment was performed by the first author (SB).

Only one eligible participant was recruited from each room in the residence hall (each room usually had 3–6 students) to prevent contamination. Moreover, about 20% of the participants were not present in the residence hall during most of the study period because it was during the semester break or the university was closed due to the COVID-19 pandemic.

### Intervention

The research team developed the educational content for eight sessions (one session per week) by reviewing all the literature, the intervention content of the previous study on this field [16], and the comments of some experts in reproductive health and psychology. The sessions' contents were mainly focused on the intervention content of the previous study in Germany [16]. For example, the general information on PMS/PMDD and the etiology were presented in the first session, while the last session included the instructions for the patients, such as the methods for relapse prevention. Both the cognitive and behavioral strategies were discussed in sessions 2 to 7. The cognitive strategies discussed in the sessions were as follows:

- Session 2: Psychological training on the role of thoughts and their relationship with emotions and behavior (cognitive triangle)
- Session 3: Changing the topic to PMS
- Session 4: Reconstruction of dysfunctional perceptions
- Session 5: Psychological training on certain superstitions about PMS and applying the cognitive strategies learned
- Session 6: Psychological training on effective thoughts and developing new assessments
- Session 7: Certain behaviors to improve PMS (use of healthcare, seeking support, and communication)

Moreover, the behavioral strategies discussed in the sessions were the following subjects:

- Session 2: Psychological training on the relationship between stress and PMS and teaching relaxation techniques
- Session 3: Psychological training on the interdependence between nutrition, exercise, and PMS
- Session 4: How to integrate exercise into daily life using a motivational program and strategy
- Session 5: What is a balanced diet and how to follow it in the daily life
- Session 6: Psychological training on the impact of stress-related errors on reasoning
- Session 7: Training on participating in positive activities in daily life

The participants were provided with audiovisual materials to help them learn practical exercises, such as relaxation techniques, exercises related to thought change, and physical exercises, including yoga.

When the educational content was prepared, the principal investigator (SB) designed a counseling course in the Learning Management System (LMS) website of the Tabriz University of Medical Sciences (Modular Object-Oriented Dynamic Learning Environment (MOODLE), https://moodle.org). The course was designed under the supervision of the person in charge (AA) of the LMS website of the university.

Special accounts were created for each member of the intervention group, and the account information was sent to them via e-mail, SMS, and one of the social media platforms they used. The intervention group received the contents in a scheduled manner. When any content was loaded on the website, the participants received an e-mail or SMS as a reminder. In addition, we created a telegram channel for them, through which we sent them daily reminders to visit the website and receive the educational content. Before the course, the participants were instructed on how to use the LMS website. Those who did not log in to their account within the first week of the course were reminded by telephone. Also, they could ask questions online and receive answers from the principal investigator (SB).

In order to check the treatment adherence, the participants were assigned homework at the end of each session and were supposed to submit their weekly feedback using the website or social media platforms. The participants with delays in sending feedback were reminded via SMS. Those who did not answer received phone calls. Also, all participants in the intervention group received calls from the principal investigator every two weeks to keep them motivated. In these calls, the principal investigator discussed the strategies with the participants, asked about the effectiveness of the strategies they used, received suggestions and criticisms, asked them to write their questions using the LMS website, and answered the questions.

The control group did not receive any intervention during the study period. After doing all post-test assessments, the accounts of the control group were created, and they received the intervention content.

### Data collection tools

We used the Persian version of the following tools to evaluate the severity of PMS symptoms, quality of life, and PMS-related disability. The tools were not under license. Moreover, the attitude toward menstruation was assessed using the Menstrual Attitude Questionnaire (MAQ). We got written permission to translate it to Persian and validate it from the original developers via e-mail.

## Researcher-made socio-demographic and reproductive questionnaire

The questionnaire was prepared using a literature review and included three parts: demographic characteristics, reproductive characteristics (including menstrual history), and contact information (Additional file 1). The face and content validity of the questionnaire was determined using the comments by ten experts.

### Daily Record of Severity of Problems (DRSP)

This questionnaire is a valid scale widely used for PMS diagnosis. It consists of 21 items on 11 areas of PMS-related symptoms (depression, anxiety, mood lability, anger, interest, concentration, lethargy, appetite, sleep, overwhelm, and physical symptoms) and three impairment items. Each item is scored on a scale of 1 (not at all) to 6 (extreme) [23]. The questionnaire must be completed for at least two consecutive cycles. The PMS is confirmed if the individual has a score of 4 (moderate) or higher in 5 or more of the symptom areas for at least two days in the late luteal phase (days − 5 to − 1 of the menstrual cycle). The score of 4 or higher should be in at least one of the items assessing depression, anxiety, affective lability, or anger/irritability, and at least one of the three impairment items. However, the individual must not have a mean score of 4 or higher in any of the symptom areas during the mid-follicular phase (days + 6 to + 10 of the menstrual cycle) [24].

In a study by Ozgoli et al., the internal consistency of the Persian version of this scale was good (Cronbach's alpha = 0.80) [25]. In the present study, the scale's reliability was also confirmed, with a Cronbach's alpha equal to 0.97 for the total scale and 0.82–0.91 for the domains. In addition, the test–retest agreement was good for the total DRSP, with an intraclass correlation coefficient (ICC) of 0.815 (95% CI 0.720 to 0.878).

### Quality of Life Enjoyment and Satisfaction Questionnaire—Short Form (Q-LES-Q-SF)

This unidimensional scale includes 14 items and assesses the quality of life during the last week. The reliability of the Persian version of this questionnaire has been confirmed in the university students in Kashan (ICC = 0.97–0.98, Cronbach's alpha = 0.93) [26] and Tabriz (Cronbach's alpha = 0.887–0.937) [8].

Women suffering from PMS have different levels of quality of life during the perimenstrual period compared to the late follicular phase of menstrual cycle [8]. Therefore, the participants filled the questionnaire twice, including once at days 1–2 and once at days 11–13 of the menstrual cycle. The internal consistency of the scale was good for both assessments (Cronbach's alpha = 0.86 and 0.85, respectively). The test–retest agreement (between the two pre-intervention assessments) was good as well (ICC = 0.783, 95% CI; 0.672 to 0.856).

### Sheehan Disability Scale (SDS)

This questionnaire is a self-report scale on disability and dysfunction [27] that evaluates the disturbances in three areas of work/school, social life, and family life/home responsibilities using a 0–10 visual analogue scale [28]. In the present study, we combined the scores of these three areas to obtain a total SDS score [29]. The Persian version of the scale has good internal consistency (Cronbach's alpha = 0.88) [28]. Its reliability in the present study was confirmed as well (Cronbach's alpha = 0.67).

### Menstrual Attitude Questionnaire (MAQ)

This questionnaire includes 33 items in 5 domains, with 5–7 items in each domain [30]. After preparing the Persian version using the forward–backward translation of the English version, its content and face validity were determined using the comments by ten experts. In the present study, its internal consistency was confirmed for all domains (Cronbach's alpha = 0.66–0.83, menstruation as a debilitating event = 0.82, menstruation as a bothersome event = 0.79, menstruation as a natural event = 0.83, anticipation and prediction of the onset of menstruation = 0.66, denial of any effect of menstruation = 0.66).

### Participant satisfaction and views on intervention effectiveness

The participants' satisfaction with the intervention was assessed using a question scored on a 5-point Likert scale (from "very satisfied" to "very dissatisfied"), while their opinions on the effectiveness of intervention were assessed using another question scored on a 6-point Likert scale (from "not at all" to "very high").

Pre-intervention assessments were already described in the sub-section of "participant recruitment." Following the 2-month intervention period, all participants in the intervention and control groups completed the DRSP during the subsequent two cycles. Moreover, they completed the Q-LES-Q-SF, SDS, and MAQ in the third month after the allocation, at the same days of the menstrual cycle that the baseline questionnaires were filled. Also, when the intervention was over, the intervention group reported their satisfaction levels with the intervention and their opinions on its effectiveness. Most questionnaires were filled online for higher accessibility.

### Data analysis

Data analysis was performed using the SPSS version 25. The normal distribution of quantitative variables was confirmed using the one-sample Kolmogorov–Smirnov test in each group. The univariate general linear models were used for intergroup comparisons of the post-intervention assessment values, which were adjusted for the baseline values. The significance level for the three primary outcomes was considered at α = 0.017 because the multiple comparisons were corrected using the Bonferroni correction method. However, the significance level for other comparisons was considered at α = 0.05. Also, we used the modified intention-to-treat (ITT) analysis and excluded the four participants lost to follow-up.

## Results

**Fig. 1:**
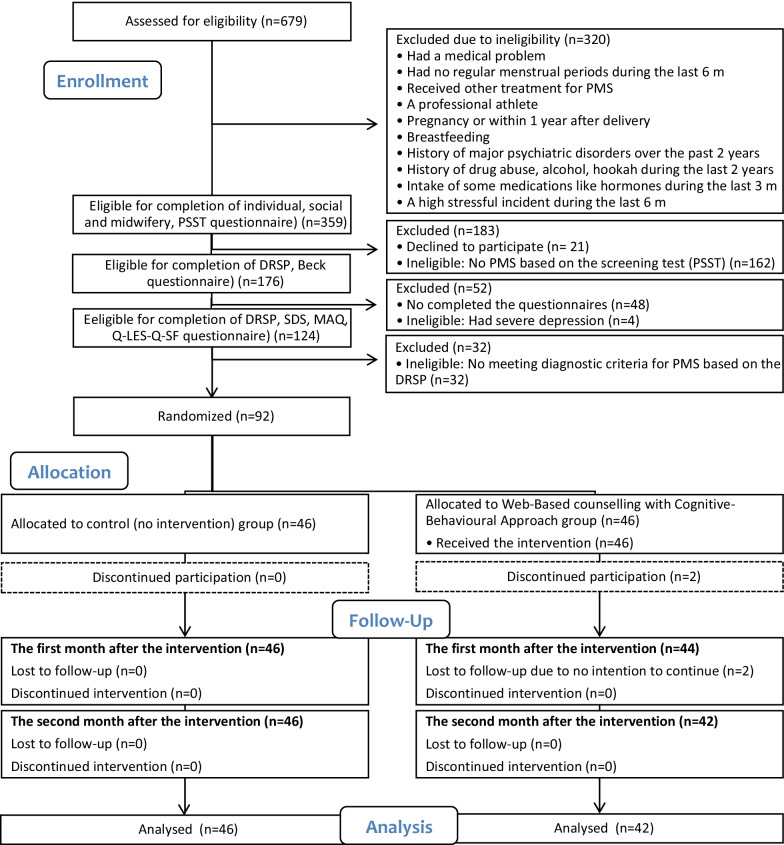
Study flow diagram. PMS, premenstrual syndrome; PSST, Premenstrual Symptoms Screening Tool; DRSP, Daily Record of Severity of Problems; SDS, Sheehan Disability Scale; MAQ, Menstrual Attitude Questionnaire; QLES-Q-SF, Quality of Life Enjoyment and Satisfaction Questionnaire Form

The participants were recruited from June 2019 to January 2020, and data collection was ended in June 2020. Of a total of 679 students screened, 92 students were eligible and agreed to participate in the study. Each group included 46 participants. Four students in the intervention group were lost to follow-up and were excluded from the analyses (Fig. 1).

The two groups were not significantly different in demographic and reproductive characteristics (Table 1), pre-test scores of symptom severity (except physical symptoms), quality of life, and attitude towards menstruation (Table 2). The participants' mean age was 22.1 years (SD 2.7) and their mean body mass index was 21.9 kg/m^2^ (SD 2.5). Moreover, 10% of the participants were married. The mean score of baseline PMS symptoms was 20.3 (SD 10.5).

**Table 1:** Baseline characteristics of participants by the study groups

Variable	Intervention (n = 46)	Control (n = 46)	P-value*
Age (years)	22.1 (2.7)	22.3 (2.5)	0.748
Menarche age (years)	12.9 (1.2)	13.0 (1.3)	0.680
PMS Score (assessed by PSST)	32.6 (6.8)	31.5 (6.4)	0.444
Depression score (assessed by Beck)	9.4 (6.9)	8.9 (7.2)	0.757
Body mass index (kg/m^2^)	22.1 (2.5)	21.6 (2.5)	0.319
Marital status (married)	5 (11%)	4 (9%)	0.726
Educational grade (master and above)	14 (30%)	16 (35%)	0.656
Family history of PMS (yes)	34 (74%)	29 (63%)	0.262
Regular exercise (yes)	10 (22%)	10 (22%)	1.000
Sufficiency of family income			
Totally	15 (33%)	21 (46%)	
To some extent	28 (61%)	23 (50%)	0.430
Not at all	3 (6%)	2 (4%)	

Intervention group received web-based counselling with cognitive-behavioural approach for eight weeks and control group received no intervention

Data present mean (SD) or number (percent)

PMS, premenstrual syndrome; PSST, Premenstrual Symptoms Screening Tool

*T-test for comparison of the means and Chi-squared test for comparison of the frequencies

**Table 2:** Comparison of the outcomes between the study groups

Outcomes	Baseline		Post-intervention			
	Intervention n = 46	Control n = 46	Intervention n = 42	Control n = 46	Adjusted difference^a^ (95% CI)	*P*
**Primary outcomes**						
Total score of premenstrual syndrome severity^b^ (0–100)	20.1 (10.7)	20.4 (10.3)	10.4 (10.0)	20.2 (10.0)	− 9.9 (− 13.3 to − 6.6)	< 0.001
Quality of life and satisfaction (0–100)^c^						
Peri-menstrual period	49.3 (16.4)	50.4 (12.8)	64.2 (15.4)	50.3 (10.8)	14.1 (8.5 to 19.8)	< 0.001
Late follicular period	63.0 (14.1)	65.5 (10.3)	68.3 (15.4)	67.3 (12.3)	1.9 (− 4.4 to 8.1)	0.975
**Secondary outcomes**						
Sub-scales of premenstrual syndrome symptoms						
Depressive symptoms (0–100)	19.1 (12.5)	18.8 (11.6)	8.7 (10.2)	18.6 (10.5)	− 10.2 (− 13.0 to − 7.3)	< 0.001
Physical symptoms (0–100)	15.1 (9.4)	20.2 (12.9)	12.7 (9.0)	18.6 (12.4)	− 2.1 (− 4.1 to − 0.1)	0.041
Anger/irritability (0–100)	26.3 (15.6)	24.2 (15.9)	12.9 (13.1)	23.7 (11.5)	− 12.0 (− 16.2 to − 7.9)	< 0.001
Reduced productivity at work/school/or home (1–6)	1.7 (0.7)	1.8 (0.7)	1.3 (0.5)	1.9 (0.7)	− 0.6 (− 0.8 to − 0.3)	< 0.001
Interfered with hobbies or social activities (1–6)	1.7 (0.7)	1.8 (0.8)	1.3 (0.5)	2.1 (0.7)	− 0.8 (− 1.0 to − 0.6)	< 0.001
Interfered with relationships with others (1–6)	1.7 (0.8)	1.9 (0.9)	1.3 (0.6)	2.3 (0.7)	− 0.9 (− 1.2 to − 0.7)	< 0.001
Disability severity^d^						
Total (0–30)	11.0 (6.2)	13.1 (4.6)	6.0 (4.7)	13.8 (5.0)	− 6.9 (− 8.7 to − 5.2)	< 0.001
In work/school work (0–10)	2.9 (2.7)	4.1 (2.4)	1.6 (1.8)	4.1 (2.5)	− 2.2 (− 3.1 to − 1.3)	< 0.001
In social life (0–10)	4.0 (2.6)	4.4 (2.0)	2.1 (1.8)	4.5 (2.0)	− 2.4 (− 3.1 to − 1.7)	< 0.001
In family life/home responsibilities (0–10)	4.1 (2.6)	4.7 (1.8)	2.3 (1.9)	5.1 (1.6)	− 2.7 (− 3.4 to − 2.0)	< 0.001
Menstrual attitude^e^						
Menstruation as a debilitating event (12–84)	59.2 (9.2)	58.9 (8.8)	52.5 (9.9)	56.9 (8.7)	− 4.7 (− 7.9 to − 1.6)	0.003
Menstruation as a bothersome event (6–42)	24.8 (6.7)	23.7 (6.1)	21.8 (6.8)	23.5 (6.6)	− 2.2 (− 4.1 to − 0.3)	0.022
Menstruation as a natural event (4–28)	25.0 (5.2)	25.7 (4.5)	27.4 (4.8)	25.4 (4.7)	2.0 (0.8 to 3.2)	0.002
Anticipation and prediction of the onset of menstruation (4–28)	26.9 (3.7)	27.4 (3.6)	25.2 (5.1)	27.0 (3.5)	− 1.5 (− 3.1 to 0.0)	0.059
Denial of any effect of menstruation (7–49)	19.8 (5.3)	19.0 (4.6)	22.3 (6.1)	18.5 (5.7)	3.3 (1.1 to 5.5)	0.004

Intervention group received web-based counselling with a cognitive-behavioural approach for eight weeks and control group received no intervention

Values indicate number (percent) or mean (SD) unless otherwise indicated

^a^Univariate General Linear Models were used to compare post-intervention scores of the groups adjusted for the baseline values using Sidak. Also, Bonferroni correction was used for the multiple comparisons of the primary outcomes

^b^Assessed using Daily Record of Severity of Problems (DRSP); the higher score, the sever symptom

^c^Assessed using Quality of Life Enjoyment and Satisfaction Questionnaire Short Form (QLES-Q-SF) twice during the period, on day of 1–2 and 11–13 of menstrual cycle; the higher score, the better quality

^d^Assessed using Sheehan Disability Scale (SDS) at day of 1–2 of menstruation period; the higher score, the sever disability

^e^Assessed using Menstrual Attitude Questionnaire (MAQ) at day of 1–2 of menstruation period

### Primary outcomes

Table 2 shows the outcomes. According to the results of the univariate general linear models adjusted for the baseline values, the mean score of total PMS symptoms was significantly lower in the intervention group than in the control group after the intervention (10.4 vs. 20.2, adjusted difference: − 9.9, 95% CI − 13.3 to − 6.6, *P* < 0.001), while the mean score of perimenstrual quality of life was significantly higher (64.2 vs. 50.3, adjusted difference; 14.1, 95% CI 8.5 to 19.8). However, there was no significant intergroup difference in the quality of life during the late follicular phase (68.3 vs. 67.3, adjusted difference: 1.9, 95% CI − 4.4 to 8.1).

### Secondary outcomes

According to the results of the univariate general linear models adjusted for the baseline values, all the sub-scales of DRSP and PMS-related disability, which was assessed using the SDS, were significantly different between the groups after the intervention (*P* < 0.001 for all, except physical symptoms with *P* = 0.041). Moreover, the mean scores of all MAQ domains were significantly better in the intervention group than in the control group after the intervention (*P* < 0.05, except the “anticipation and prediction of the onset of menstruation” with *P* = 0.059, Table 2). Also, the single questions scored based on the Likert scale indicated that all students of the intervention group were satisfied with the ICBT course and declared that their symptoms had improved post-intervention.

## Discussion

According to our results, ICBT significantly reduced the severity of PMS-related symptoms and PMS-related disability, while it improved the quality of life during the premenstrual period (days − 5 to + 2 of the menstrual cycle) and attitude toward menstruation in the female university students with PMS. However, it did not significantly affect the quality of life in the late follicular phase (about days + 5 to + 12 of the menstrual cycle).

Our results on the effectiveness of ICBT on PMS-related symptoms are compatible with the similar trial conducted in Germany on women with PMDD [31]. Other trials conducted in Iran also reported the effectiveness of internet-based programs on reducing the severity of PMS symptoms [32, 33] and on improving general health of the affected individuals [33]. However, the approaches used and intervention duration were not reported in these studies. Also, another trial in Iran [34] showed the improving effect of 10 weekly sessions of cognitive-behavioral group therapy with the duration of 90 min. The sessions were not internet-based and were held by two clinical psychologists for the students suffering from PMS in order to improve their health-related quality of life.

The effectiveness of ICBT on PMS patients can partly be explained by the efficacy of this intervention in changing the attitudes of the affected individuals toward menstruation. A study in Turkey showed that the women suffering from PMS had a significantly higher score in the debilitation subscale and a significantly lower score in the denial subscale of the MAQ than the women without PMS [35]. We also observed that this intervention could improve the scores of the patients in the MAQ subscales, thereby improving their attitudes toward menstruation.

The significant effect of the study intervention on quality of life during the perimenstrual period, as well as its insignificant effect on the quality of life during the late follicular phase, seems logical because the intervention could reduce the severity of premenstrual symptoms, resulting in improved quality of life during the related period. Consistent with the results of another study conducted by the same team [8], in this study, the baseline mean score of quality of life was already significantly higher in the late follicular period than in the perimenstrual period.

Due to the nature of the study, it was not possible to blind the participants, healthcare providers, and outcome assessors, who were also the participants. However, it seems that the risk of different biases was low. Achieving consistent results using several validated and prospective tools for consequence assessment and the insignificant effect of the intervention on the quality of life during the late follicular phase suggested the low level of detection bias in the present study. Also, we had a few participants lost to follow-up. Therefore, the plausible effect size was insufficient to exert a clinically relevant effect on the outcomes. Thus, the risk of attrition bias was low as well.

Contrary to our previous plans, we could not recruit any participants from the private residence halls due to the lack of cooperation of the persons in charge. However, the participant allocation was properly randomized and concealed, so there was no selection bias. Also, excluding the students in private residence halls had little or no effect on the generalizability of the results because just a small percentage (15%) of students lived in the private residence halls, and their clinical and demographic characteristics were not significantly different from those living in public residence halls.

We tried to minimize contamination by recruiting only one person from each room in the residence halls, creating a separate account for each participant in the intervention group, and notifying them not to share the content with others until the end of the study.

Given the obtained results, including the intervention effectiveness, high acceptability of the method, low attrition, and high levels of participant satisfaction with the intervention, it seems that the ICBT can be used as a suitable non-pharmacological option for alleviating the PMS symptoms and improving the quality of life of the sufferers. More than 80% of the world's adult population are literate [36], with about 50% of the female population having access to the internet [37]. The data of the 2016 census in Iran showed that 94% of Iranian women of reproductive age were literate, and 55% had 12 years of education (27%) or higher (28%) [38]. Moreover, two-thirds (66%) of Iranian women used the internet [39], and almost 80% of households had access to the internet [40]. Therefore, most women suffering from PMS throughout the world, including Iran, can use internet-based therapies.

Internet-based therapies have some benefits over face-to-face counseling sessions. For example, there is no need for the patients to attend a therapy session in a specific location. Thus, the traveling expenses and time waste are reduced [41–43]. The educational content is available 24 h a day [44], and the participants can review the contents several times so that the possibility of forgetting the learned topics is minimized [45]. It encourages self-care [46] and provides the possibility of confidential communication with the system administrator [44, 47].

We did not follow the participants more than two menstrual cycles after the end of the intervention. Thus, it is not possible to comment on the long-term effectiveness of the intervention. In addition, the study participants included only medical students, so results may not be generalized to other populations. Therefore, it is suggested to conduct further studies with longer follow-up durations on other populations, such as students in other fields, non-student women, or women of different ages. Also, we recommend investigating the impact of such interventions on the quality of life of the family members, such as husbands, of these women in future studies.

## Conclusions

The present study showed that ICBT could reduce the severity of PMS symptoms and improve the quality of life of the affected individuals during the perimenstrual period. However, it had no significant effect on the quality of life during the late follicular phase. In addition, this intervention could improve the attitudes toward menstruation. It is recommended that health policymakers and managers develop policies to use ICBT for women suffering from PMS, especially in situations like the Covid-19 pandemic.

## Supplementary Information

**Additional file 1:** Researcher-made demographic and reproductive questionnaire

## Data Availability

The datasets used and/or analyzed during the present study are available from the corresponding author on reasonable request.

## Abbreviations

PMS: Premenstrual syndrome
ICBT: Internet-based cognitive-behavioral therapy
DRSP: Daily Record of Severity of Problems
Q-LES-Q-SF: Quality of Life Enjoyment and Satisfaction Questionnaire—Short Form
PMDD: Premenstrual dysphoric disorder
PSST: Premenstrual Symptoms Screening Tool
SDS: Sheehan Disability Scale
MAQ: Menstrual Attitude Questionnaire
ICC: Intraclass correlation coefficient
