# Supplementary material for: Internet-based cognitive-behavioral therapy for premenstrual syndrome: a randomized controlled trial
Source: BMC Womens Health. 2022 Jan 8;22:5. doi: 10.1186/s12905-021-01589-7 (PMC8741531; doi:10.1186/s12905-021-01589-7)
Supplement: Supplementary file 1 — Additional file 1: Researcher-made demographic and reproductive questionnaire [file 12905_2021_1589_MOESM1_ESM.docx]

| Demographic-social and Reproductive Characteristics Questionnaire |
| --- |

**Code /study number:** ................................

**Dear student,**

Please read all the questions **in order** and **carefully** and enter your answers in the designated place. Please do not leave any question unanswered.

**Personal Information**

1. Year of Birth: …………………. 2. Province of birth: ……………….
2. Study grade: Associate ⃝ Bachelor ⃝ Master ⃝ Doctorate ⃝ Ph.D ⃝
3. Field of Study: …………………. 5. Year of university entry: ……………

6. Weight (kg): ………….. 7. Height (cm): ……

8. Are you employed? No ⃝ Yes ⃝, please mention your job ……………

9. Marital status: Single ⃝, Please jump to the question 10.

Married ⃝, If married, please answer the followng questions

- 1. Year of marriage: …………., b. Year of birth of husband ………..

1. Educational level of husband …………….
2. Gravida: ……. e. Number of children: ….. f. Number of abortions: ….
3. Adequacy of monthly household income for living expenses:

Quite sufficient ⃝ Somewhat sufficient ⃝ Not sufficient at all ⃝

11. Do you exercise regularly? (Regular exercise activities means doing various sports exercises such as walking, etc. for at least half an hour to an hour and 3 to 4 times a week.)

No ⃝ Yes ⃝, If yes, please answer the following questions

How many times a week do you exercise on average? ....................... times

How many minutes do you exercise on average each time? ........................ minutes

12. Have you taken any medicine in the last 2 months?

No ⃝, Yes ⃝, If yes, drug/s name: .................................

**History of menstrual cycles and premenstrual syndrome**

13. Age of first menstruation: ……. Years

14. Average duration of menstural cycle in the last 3 cycles (each menstural cycle start from the start of your mensturation to the start of your next mensturation): …….. days

15. Are there any signs of premenstrual syndrome among your family members (mother, sister, etc.): No ⃝, Yes ⃝, If yes, mention her relation with you: .................................

16. How long have you been experiencing the symptoms of premenstrual syndrome:

…….. years, ….. months

1. Have you seen a doctor in the last 2 months because of premenstrual symptoms?

Yes ⃝ No ⃝

1. Have you attended counseling sessions in the last 2 months due to premenstrual symptoms? Yes ⃝ No ⃝
2.
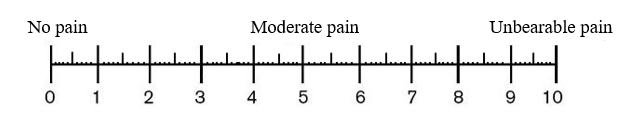
In the picture below, If the number 10 is the unbearable pain, the number zero is no pain and the number five is moderate pain, considering the intensity of the pain you experienced during the last three menstrual cycles during menstrual bleeding, which number will be your menstrual pain?

**Contact information**

1. Phone number: ……………………… 21. Email: ………………………………………..
2. Do you have a personal computer: Yes ⃝ No ⃝
3. Can your mobile phone connect to the internet: Yes ⃝ No ⃝
4. Do you have an account on social networks: Yes ⃝ No ⃝
5. What social networks are you a member? (Please mention your user ID)

Telegram ⃝, user ID ………………. WhatsApp, user ID ………………

Others ⃝, please mention the name ………….. & User ID ………………..

1. Do you have internet access at your place of residence: Yes ⃝ No ⃝
2. How much do you estimate your average internet usage:

…………….hours per day ………….. minutes per day I do not use it daily ⃝
